# Current perspective on retinal remodeling: Implications for therapeutics

**DOI:** 10.3389/fnana.2022.1099348

**Published:** 2022-12-22

**Authors:** Rebecca L. Pfeiffer, Bryan W. Jones

**Affiliations:** Moran Eye Center, Department of Ophthalmology, University of Utah, Salt Lake City, UT, United States

**Keywords:** retinal remodeling, neurodegeneration, therapeutics, age-related macular degeneration, retinitis pigmentosa

## Abstract

The retinal degenerative diseases retinitis pigmentosa and age-related macular degeneration are a leading cause of irreversible vision loss. Both present with progressive photoreceptor degeneration that is further complicated by processes of retinal remodeling. In this perspective, we discuss the current state of the field of retinal remodeling and its implications for vision-restoring therapeutics currently in development. Here, we discuss the challenges and pitfalls retinal remodeling poses for each therapeutic strategy under the premise that understanding the features of retinal remodeling in totality will provide a basic framework with which therapeutics can interface. Additionally, we discuss the potential for approaching therapeutics using a combined strategy of using diffusible molecules in tandem with other vision-restoring therapeutics. We end by discussing the potential of the retina and retinal remodeling as a model system for more broadly understanding the progression of neurodegeneration across the central nervous system.

## Introduction

Retinal degenerative diseases (RDDs) impact millions of people. Age-related macular degeneration (AMD) currently impacts 196 million people worldwide and is projected to nearly double by 2050 (Wong et al., 2014). This expected increase in AMD is due to a greater portion of the global population exceeding 65 years old when the probability of AMD becomes much higher (Lim et al., 2012). AMD is by far the most prevalent RDD; however, its root cause, particularly the dry form of AMD, is not entirely clear. We know that genetics combined with environmental factors are strongly associated with the development of AMD, presenting difficulty in its study (Al-Zamil and Yassin, 2017). Although other RDDs like retinitis pigmentosa (RP) are less prevalent, RP affects 1 in 4,000 people and typically onsets early in life (Hartong et al., 2006). RP is associated with many specific genetic defects, enabling the identification and creation of numerous naturally occurring or transgenic animal models (Keeler, 1924; Aguirre, 1978; Narfstrom, 1983; Barnett and Curtis, 1985; Pittler et al., 1993; Suber et al., 1993; Brockerhoff et al., 1995; Petters et al., 1997; Semple-Rowland and Lee, 2000; Chang et al., 2002; Kondo et al., 2009; Han et al., 2013; Chow et al., 2015). These RP models are used in conjunction with donor tissue from patients with RP and AMD to understand the progression and downstream effects of photoreceptor degeneration, revealing fundamental changes to neuronal and glial populations (Strettoi and Pignatelli, 2000; Strettoi et al., 2002, 2003; Jones et al., 2003, 2011, 2016a,b; Marc and Jones, 2003; Pfeiffer et al., 2020b). Due to this pressing public health concern, an increasing number of research groups are searching for therapies to stop or reverse the photoreceptor loss and retinal damage associated with AMD and RP. Additionally, many groups are working to restore light perception following the loss of photoreceptors in more advanced cases of retinal degeneration (Yue et al., 2016; Menon and Vijayavenkataraman, 2022).

Here we provide a perspective of the fundamental progression of retinal remodeling and its impacts on some of the more common approaches being investigated for recovery of vision loss. We will conclude with a discussion of some promising molecular strategies for neuroprotection that, when used in conjunction with vision rescue techniques, may prove to be instrumental in overcoming the obstacles generated by retinal remodeling.

## Retinal remodeling

Photoreceptor loss and subsequent retinal remodeling are caused by numerous primary mechanisms, including RDDs (described above), or caused by injuries such as light-induced damage, retinal detachment, or ischemia (Erickson et al., 1983; Lewis et al., 1989, 1991; Jones et al., 2006; Krigel et al., 2016). It has been noted for nearly a century that photoreceptor degeneration leads to several negative plasticity events. In 2003, these observations were consolidated into the term: retinal remodeling and broadly divided into 3 phases and ending with widespread neurodegeneration (Jones et al., 2003; Marc and Jones, 2003).

### Phase 1

**FIGURE 1:**
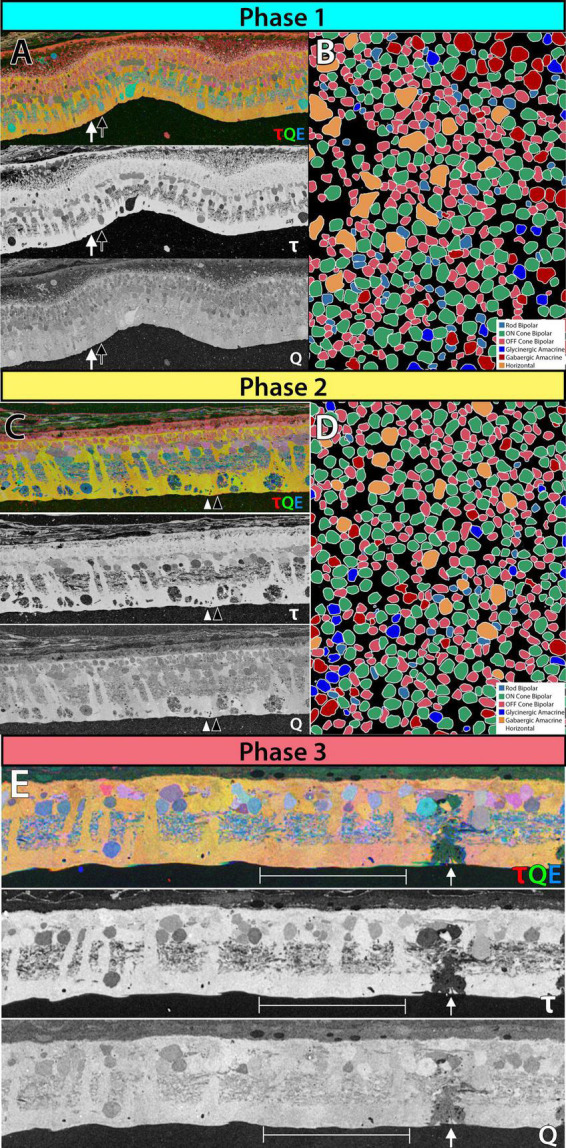
Metabolic and receptor changes phase 1 remodeling in 3mo P347L rabbit: **(A)** top panel: 3-channel composite of metabolites taurine, glutamine and glutamate. Lower panels: individual metabolites taurine and glutamine. Arrows indicate neighboring Müller cells. **(B)** Theme map of cell types in a horizontal section of retina identified based on ionotropic glutamate receptor function. Phase 2 remodeling in 2yo P347L rabbit: **(C)** top panel: 3-channel composite of metabolites taurine, glutamine and glutamate. Lower panels: individual metabolites taurine and glutamine. Arrows indicate neighboring Müller cells. **(D)** Theme map of cell types in a horizontal section of retina identified based on ionotropic glutamate receptor function. Phase 3 remodeling in 4yo P347L rabbit: **(E)** top panel: 3-channel composite of metabolites taurine, glutamine, and glutamate. Lower panels: individual metabolites taurine and glutamine. Arrows indicate Müller cell devoid of taurine and containing reduced glutamine. Bracket indicates region of Müller cells with varying levels of small molecules.

**FIGURE 2:**
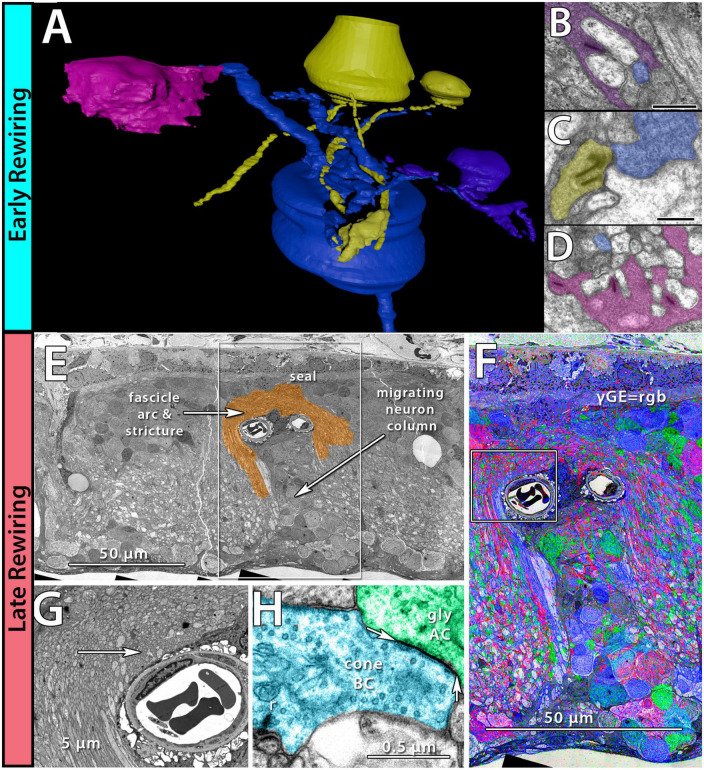
Early and late-stage rewiring. Early rewiring in 10mo P347L rabbit: **(A)** 3D rendering of rod bipolar cell (RodBC) dendrites from RPC1. RodBC in blue, cone photoreceptor in pink, rod photoreceptor in purple, and indeterminate in yellow. **(B)** Pseudocolored TEM image of rod photoreceptor ribbon synapse onto RodBC rendered in panel **(A)**. **(C)** Pseudocolored TEM image of indeterminate photoreceptor ribbon synapse onto RodBC rendered in panel **(A)**. **(D)** Pseudocolored TEM image of Cone photoreceptor ribbon synapse onto RodBC rendered in panel **(A)**. Scale bars: 500 nm late rewiring in the pnd 900 RCS rat: **(E)** TEM image of microneuroma extended over a blood vessel invading inner nuclear layer. **(F)** GABA, Glycine, and Glutamate channels overlayed on top of the region outlined with a box in panel **(E)**. **(G)** Higher resolution image of region highlighted with box in panel **(F)**. **(H)** Pseudocolored high-resolution image of area indicated with arrow in panel **(G)**. Arrows indicate edges of gap junction found intact within microneuroma.

Phase 1 remodeling is characterized by the initiation of photoreceptor stress and degeneration. The impact of photoreceptor degeneration upon the retina, even in the earliest stages, is not limited to photoreceptors. Glial cells rapidly respond to photoreceptor stress through the activation and interaction of microglia and Müller cells (Jones et al., 2003, 2011, 2014; Pfeiffer et al., 2016; Di Pierdomenico et al., 2020). In phase 1 remodeling, microglia invade the retina, contributing to photoreceptor degeneration (Peng et al., 2014; Di Pierdomenico et al., 2019), while Müller cells begin to hypertrophy and alter their metabolic signature, showing variability in concentrations of taurine and glutamine (Figure 1A). Changes in taurine levels are a proposed mechanism contributing to retinal degeneration (Garcia-Ayuso et al., 2019; Di Pierdomenico et al., 2022; Martinez-Vacas et al., 2022). Simultaneously, there is a pharmacologic and/or protein expression shift in bipolar cells demonstrating increased responses consistent with ionotropic glutamate receptors (Marc et al., 2007; Chua et al., 2009; Jones et al., 2011), generally associated with the OFF bipolar cell class (Figure 1B). Based on more recent work in pathoconnectomics we see substantial inner retinal rewiring (Pfeiffer et al., 2020). Rod bipolar cells (RodBCs), demonstrate synaptic connectivity with cone photoreceptor pedicles and neurite outgrowths off of cone photoreceptors (Figures 2A–D). These novel connections are made while many of the RodBCs still maintain some degree of connectivity with surviving rod photoreceptors, creating complex bipolar cell input paradigms. In the inner plexiform layer, RodBCs make gap junctions with their primary postsynaptic partner, the Aii amacrine cell. This phenomenon is never observed in the normal, healthy retina, potentially explaining the reports of poor adaptation by RP patients early in the course of their disease. Beyond immediate partner rewiring, GABAergic amacrine cells project from the inner nuclear layer up into the outer plexiform layer, making morphologically identifiable synapses with horizontal cells, and bipolar cell dendrites. This topology is also entirely novel and pathological as it introduces network corruption in the visual pathway.

In summary, phase 1 remodeling demonstrates glial activation and widespread rewiring or retinal network revision occurs early in degeneration, potentially complicating many therapeutic interventions.

### Phase 2

Prolonged photoreceptor degeneration characterizes phase 2 remodeling. Here, glial activation becomes increasingly pronounced through microglial migration (Di Pierdomenico et al., 2017), greater metabolic variability (Figure 1C), morphological entanglement of Müller cell endfeet (Pfeiffer et al., 2019), beginning of the formation of the Müller seal (Jones and Marc, 2005; Pfeiffer et al., 2016; Pfeiffer et al., 2019), and upregulation of proteins such as GFAP (Erickson et al., 1987). Reprogramming continues with the mislocation of mGLuR6 receptors away from ON-BC dendrites (Nomura et al., 1994; Chua et al., 2009), and ionotropic receptor consistent responses in bipolar cell population increase (Figure 1D). Rewiring also becomes more pronounced through the extension of large neurites from horizontal cells into the inner plexiform layer (Strettoi and Pignatelli, 2000; Strettoi et al., 2002, 2003). Remaining photoreceptors may also extend neurites beyond the outer plexiform layer into the inner plexiform layer or into the ganglion cell layer (Li et al., 1995; Fariss et al., 2000; Sethi et al., 2005). Additionally, some bipolar cells retract their dendrites completely (Strettoi and Pignatelli, 2000; Strettoi et al., 2003). The total effects of rewiring on the inner plexiform networks and the implications for current flow through the altered network are currently unknown. However, the investigation into pathoconnectome volumes will provide a framework to begin hanging gene and protein expression data, as well as providing complete networks for modeling. At the end of Phase 2, the last of the rod photoreceptor somas disappear, and most of the cone somas are also gone. Some rare cone photoreceptor somas may persist, underneath which there may be localized areas of some retinal preservation, but remodeling is progressing regardless.

### Phase 3

The complete loss of photoreceptor somas characterizes phase 3 remodeling. The retina engages in a prolonged phase of revision, potentially driven by Müller cell gliosis. Phase 3 remodeling ultimately renders the retina unrecognizable as it transitions into the neurodegeneration phase. Glial metabolic variability persists (Figure 1E) while structural alterations seal off the neural retina through a Müller cell seal and increasing entanglement of Müller cell processes (Lewis et al., 1989; Pfeiffer et al., 2020a,b). Unlike the glutamate receptor reprogramming seen in phases 1 and 2, by phase 3, AGB loading shows glutamate receptors to be functionally or pharmacologically absent in remaining bipolar cells (Marc et al., 2007). Gross rewiring is prevalent throughout the retina, with neurite sprouts from all remaining neuronal cell classes coalescing into tangles called microneuromas (Figures 2E–H). Within microneuromas, neurites form synapses with ultrastructure consistent with numerous synaptic types. However, the pairing of these and whether they follow network rules similar to the healthy retina is unknown (Jones et al., 2003; Jones and Marc, 2005). Our pathoconnectomics initiatives are actively exploring this question. Ultimately, loss of inner retinal neurons devolves into a complete neurodegenerative phenotype, though there is not a clear aspect that delineates the neurodegenerative phase from phase 3 remodeling.

### Widespread neurodegeneration

If retinal remodeling persists long enough, the retina will eventually lose upward of 90% of its neurons (Pfeiffer et al., 2020b). At this point, even ganglion cells, previously reported to be spared from neurodegeneration in RDDs (Medeiros and Curcio, 2001; Stefanov et al., 2019), also degenerate (Garcia-Ayuso et al., 2015, 2018). This is the most severe outcome, occurring only after prolonged remodeling. However, as people live longer, we may see more instances of widespread retinal degeneration in patients, particularly given the increase in diabetic retinopathy and AMD projected to increase substantially over the next two decades.

## Retinal degeneration therapies

### Genetic therapies

Genetic intervention is undoubtedly one of the therapeutic interventions most heavily invested in Botto et al. (2022). Intervening genetically prior to the degeneration of neurons would be ideal to avoid many complications of remodeling (Jones et al., 2016a,b; Pfeiffer et al., 2020b), but it faces many logistical challenges. That said, the genetic approach has seen clinical success, particularly in Luxterna, the *RPE65* genetic therapy for treating Leber’s Congenital Amaurosis (Acland et al., 2001; Le Meur et al., 2007; Maguire et al., 2021). Numerous other genetic therapies are currently being developed based on this success (Prado et al., 2020). However, initial successes in treatment were followed by the progression of visual deficits afterward, suggesting that the retinal remodeling processes, initiated prior to gene therapy, is an ongoing program not arrested by this particular gene therapy intervention. This would certainly track with our failed optogenetic study (unreported) in rabbits that did not stop the process of retinal remodeling.

In principle, treating neurodegenerative diseases prior to the damage of the initially affected neurons is ideal. In practice, this would be effective on single genetic cause diseases, not complex diseases, including AMD. When not suspected due to familial history, even single genetic cause disorders may have substantial neurodegeneration before diagnosis, and the initiation of retinal remodeling is likely underway. Realistically, photoreceptor degeneration diseases are associated with roughly 300 known primary mutations (Daiger et al., 2014), making it challenging to produce a novel therapeutic for each genetic cause, as each gene defect is effectively an orphan disease. There may also be more gene-gene or gene-environment disorders within the constellation of RDD than we can realistically generate genetic therapies for. Combined, this demonstrates a need for further understanding of remodeling processes.

### Optogenetics

The field of optogenetics allows control or activation of cells using light (Nagel et al., 2003; Boyden et al., 2005). Optogenetic therapies have been proposed for classic neurodegenerative diseases such as Alzheimer’s and Parkinson’s disease, and perhaps more intuitively for retinitis pigmentosa to restore light-sensing cells to the retina following photoreceptor degeneration (Marc et al., 2014). Early clinical trials for optogenetics usage in retinitis pigmentosa are coming out with encouraging results (Sengupta et al., 2016; Sahel et al., 2021). However, as highlighted by Harris and Gilbert (2022), even promising studies are not without potential concern. The usage of AAVs to deliver photosensitive proteins may cause future complications in treatment, particularly in the potential for an immune response to AAVs as they become more therapeutically widespread. Additionally, the permanent nature of optogenetic interventions essentially removes the ability to stop or reverse treatment should the treatment prove long-term to have adverse effects. Therefore, although optogenetic treatments demonstrate great promise for the treatment of RDDs, clinical trials should proceed with caution. Within the context of retinal remodeling, optogenetic interventions are complicated by the early rewiring and glutamate receptor reprogramming of bipolar cells, causing conflicting input to ganglion cells. Appropriate targeting to the precise class of cells is also potentially problematic. With >40 classes of ganglion cells, which ganglion cell classes will we target for gene therapy? Also, what are the psychophysics of introducing additional sensory percepts within a normal retinal network? What happens when that network is altered as it is in retinal degeneration and remodeling?

### Photoswitches

Another mechanism to induce photo-sensitivity in neurons that are not innately photosensitive is through the use of photoswitches (Kramer et al., 2009; Polosukhina et al., 2012; Marc et al., 2014; Tochitsky et al., 2014). Photoswitches are photosensitive small molecules which reversibly alter their conformation in response to light, conferring a light response to the cells they are interacting with (Szymanski et al., 2013). Photoswitches are easily applied through simple intravitreal injection and are reported to be non-toxic. Many of the early generations of photoswitches interact with ion channels allowing direct control of the channel state by light, blocked or unblocked (Kramer et al., 2009). Newer photoswitches have been engineered to create light-inducible forms of glutamate receptors, ionotropic, or metabotropic, allowing a more direct recapitulation of intact retinal function. One primary concern is that these compounds appear to be most effective in non-light sensing retinas (Tochitsky et al., 2014), although preservation of vision requires intervention earlier in degeneration. Despite the promising results of early studies of these compounds in animals, more research is needed prior to use in the clinic. The use of photoswitches in RDD is also complicated by rewiring and reprogramming, but the temporary nature of this intervention allows for more trial and error in their implementation.

### Cell replacement

Over the last decade, our capabilities differentiating stem cells into specific neural lineages have increased dramatically (Yamanaka, 2020). This advancement has led to renewed interest in replacing damaged neurons in neurodegenerative diseases (Gagliardi et al., 2019). Photoreceptor degenerations are especially attractive for this therapy because of the nature of disease progression, namely the initial damage of the photoreceptors, their subsequent loss, and the ease of accessing the retina compared with other CNS regions. Additionally, the success of culturing retinal organoids has led to a diverse array of available retinal neural types, including photoreceptors (Cowan et al., 2020; Li et al., 2021). Cell replacement therapy as a discipline is still in its infancy, leaving many technical challenges and questions involving correct circuit integration within an altered network, long-term survival of the transplanted cells (Wu et al., 2018), and impacts on native cells (Zhou et al., 2021) to be explored. Another related approach is the dedifferentiation of Müller glia into stem cells for an endogenous approach to cell replacement (Lamba et al., 2009; Hoang et al., 2020). However, its implementation is hindered by cell specificity in Müller cell targeting (Le et al., 2022) and the same circuit integration complications as neuronal transplants. That said, Müller cells are ideally positioned within the retina, and would also have intimate contact with other Müller cell populations as they de-differentiate to maintain access to Müller cells’ retinal metabolic support.

### Bionics

The use of electrode implants to restore vision has advanced considerably (Weiland et al., 2011; Chuang et al., 2014; Stingl et al., 2017; Farnum and Pelled, 2020). A primary advantage of implants is the ability to select their placement depending on the state of retinal degeneration. If the inner retinal circuitry is still largely intact, a subretinal implant may be most effective at restoring more acute vision by utilizing the inner retinal processing (Zrenner, 2002). Alternatively, should the inner retinal wiring be corrupted through remodeling processes, ganglion cells serve as a potential target through epi-retinal implants. Lastly, implants can also be engineered to bypass the degenerating retina and directly interface with the cortex. Clinical trials of all implants demonstrate early successes in restoring some vision to patients who were previously non-light perceiving (Ahuja et al., 2011; Dorn et al., 2013; Beauchamp et al., 2020); however, movement of the implant, long-term efficacy, and sustained support for permanent implants are ongoing concerns (Kuehlewein et al., 2019). Despite the early successes of bionics, all implants are longitudinally impacted by issues of device movement and gliosis. More broadly, retinal bionic implants have not been shown to slow down or reverse the retinal plasticity associated with retinal degeneration. To address some of these concerns, modeling groups have begun exploring how remodeled retinal networks respond to electrical stimulation in contrast to the responses predicted from a healthy retina (Kosta et al., 2021).

### Diffusible molecules and neuroprotection

The extensive inner retinal damage and complications for therapeutic interventions associated with retinal remodeling can seem overwhelming. However, when combined with current neuroprotective strategies, many avenues of treatment may prove effective in the retina. This is not an exhaustive list of neuroprotective strategies, and we refer readers to some excellent reviews (Froger et al., 2014; Kolomeyer and Zarbin, 2014; Vecino et al., 2016; Pardue and Allen, 2018).

One active area of exploration for neuroprotection is in releasable signaling molecules like dopamine. Dopamine released from dopaminergic amacrine cells plays important roles in light adaptation and signal adaptation (Witkovsky, 2004). It is also implicated in the progression of numerous neurodegenerative diseases, including Parkinson’s disease, diabetic retinopathy, and may play a role in AMD (Mor et al., 2019; Figueroa et al., 2021). Therapeutically, dopamine analogs like L-DOPA or dopamine agonists have been used for a long time to treat Parkinson’s disease to counteract the loss of dopaminergic neurons in the brain. In the retina, the loss of dopaminergic neurons coincides with disease progression and experimental treatment paradigms in animal models are promising (Ivanova et al., 2016). In humans, L-DOPA administration in Parkinson’s patients potentially delays the onset of AMD, though this could be acting through a downstream target (Brilliant et al., 2016). These results indicate that dopamine may be critical for the function and survival of many neuronal classes across the CNS and warrants further study within the realm of neuroprotection.

Another potential molecule class to combat neurodegeneration are the steroid hormones progesterone and estrogen. Progesterone and estrogen have been implicated as neuroprotectants in various CNS insults, including traumatic brain injury, and experimentally in light-induced retinal degeneration (Attella et al., 1987; Zhu et al., 2015). However, despite promising animal studies, progesterone treatments have not yet demonstrated efficacy in clinical trials (Sitruk-Ware et al., 2021). The mechanism of action leading to neuroprotection by progesterone and estrogen is not entirely clear. They both act on pathways inducing decreasing microglial activation, decreasing cytokine activation, increasing levels of brain-derived neurotrophic factor (BDNF), acting as an agonist of GABA receptors, and reducing edema (Singh and Su, 2013; Roche et al., 2016; Guennoun, 2020). These roles could be useful for managing numerous retinal and brain neurodegenerative diseases, and warrant further exploration.

Finally, one of the more commonly explored neuroprotective strategies is the direct modulation of neurotrophic factors (Garcia et al., 2003). There are two primary families of neurotropic factors: glial-derived neurotrophic factors (GDNF) and neurotrophins which include BDNF, nerve growth factor (NGF), and neurotrophin 3 and 4 (NT3 and NT4) (Bringmann et al., 2009). Neurotrophic factors have important roles in neuronal development and survival (Hodgetts and Harvey, 2017; Skaper, 2018). Many neurotrophic factors are predominately released by glial cells and work through multiple pathways to modulate synaptic strength, promote neuronal survival, and are involved in neurite outgrowth during development. The potential of neurotropic factors for treating neurodegenerative diseases has not gone unnoticed (Rangasamy et al., 2010; Johnson et al., 2011). However, their hydrophilic properties has complicated their delivery (Thorne and Frey, 2001). Despite this hurdle, experimental usage *in vitro* and in animal models is encouraging and neurotrophic factors may prove to be an important component of neurodegenerative disease treatment in the brain and retina (Allen et al., 2013).

## Discussion

Retinal degenerative diseases such as RP and AMD are debilitating diseases leading to progressive vision loss and eventual neurodegeneration of the inner retina. Although the progressive nature of these diseases and the accompanying retinal remodeling complicate most approaches to therapeutic interventions, there is substantial hope for treatment including through the evaluation of remodeling processes themselves (Peng et al., 2014; Di Pierdomenico et al., 2018, 2019). Additionally, many aspects of retinal remodeling and degeneration recapitulate components observed in other CNS neurodegenerations (Pfeiffer et al., 2020b). These observations open a new avenue of exploration into fundamental mechanisms of neurodegeneration potentially conserved across the nervous system. The retina is ideal for evaluating these mechanisms because of its compact size, immune privilege, entire circuit topologies, and the accessibility for non-invasive monitoring. Combined, it is our hypothesis that key treatments for neurodegenerative diseases will be combinations of engineering approaches like cell replacement combined with molecular interventions of diffusible molecules to delay or prevent neuronal loss and deleterious remodeling.

## Data availability statement

The original contributions presented in this study are included in this article/supplementary material, further inquiries can be directed to the corresponding authors.

## Ethics statement

The animal study was reviewed and approved by the University of Utah IACUC.

## Author contributions

Both authors wrote the perspective and contributed panels to the figures.
